# Editorial: Community series in primary immunodeficiencies worldwide, volume III

**DOI:** 10.3389/fimmu.2026.1913475

**Published:** 2026-06-30

**Authors:** Hirokazu Kanegane, Antonio Condino-Neto, Jolan E. Walter

**Affiliations:** 1 Department of Child Health and Development, Graduate School of Medical and Dental Science, Institute of Science Tokyo, Tokyo, Japan; 2 The Jeffrey Modell Center for Primary Immunodeficiencies São Paulo, São, Paulo, Brazil; 3 Division of Pediatric Allergy and Immunology, Department of Pediatrics, University of South Florida, St., Petersburg, FL, United States; 4 Johns Hopkins All Children’s Hospital Children’s Research Institute, St., Petersburg, FL, United States

**Keywords:** autoimmunity, immune dysregulation, inborn errors of immunity, newborn screening, primary immunodeficiencies

## Introduction

Primary immunodeficiencies (PIDs), also known as inborn errors of immunity (IEIs), are rare disorders characterized by impaired immune function, resulting in increased susceptibility to infections and immune dysregulation. This research topic summarizes key findings from articles published in Volume III of the “Community Series in Primary Immunodeficiencies Worldwide” and aims to deepen understanding of PIDs while supporting improvements in their diagnosis and treatment.

## Mechanisms and conceptual framework of the disease

PID/IEI was once considered a disease characterized solely by susceptibility to infection. However, more than 500 diseases have now been identified, with autoimmune diseases and immune dysregulation as established as frequent manifestations.

Elamine et al. note that autoimmune symptoms may preceed infections and serve as early indicators of underlying PID/IEI. They identify early-onset autoimmune diseases, the coexistence of multiple autoimmune diseases, refractory or atypical disease courses, and the development of autoimmune diseases following infection as “red flags” for suspected PID/IEI.

Furthermore, four mechanisms have been proposed to contribute to the breakdown of immune tolerance. These include failure to eliminate autoreactive clones because of defects in central immune tolerance, accumulation of autoreactive lymphocytes resulting from impaired lymphocyte apoptosis, reduced numbers and impaired function of regulatory T cells, and increased survival of autoreactive B cells due to dysregulation of B-cell activating factor and a proliferation-inducing ligand. Understanding these mechanisms may allow individualized disease assessment and the identification of therapeutic targets.

## Diagnostic approaches and epidemiology

This discussion highlights both comprehensive screening and retrospective diagnostic approaches. Boyarchuk et al. reported findings from a newborn screening (NBS) program in Ukraine. Between 2022 and 2025, 398,415 newborns underwent NBS. Abnormalities in T-cell receptor excision circles and/or kappa-deleting recombination excision circles were detected in 57 cases, and 18 were subsequently diagnosed with PID/IEI, including 7 with severe combined immunodeficiency (SCID). The incidence of SCID was 1:49,800, comparable to rates in other countries. The survival rate following hematopoietic stem cell transplantation (HSCT) was 85.7%.

Nishimura et al. described a case in which a preserved umbilical cord facilitated retrospective diagnosis and clarification of family history. To determine the cause of an unexplained fatal infection during infancy, the investigators extracted DNA from a preserved dried umbilical cord—a practice rooted in Japanese cultural tradition—and performed genetic testing. The analysis revealed that a deceased uncle in each of two families had X-linked agammaglobulinemia.

Hereditary angioedema (HAE) is characterized by recurrent episodes of localized edema caused by excessive bradykinin production. Kurjane et al. investigated the prevalence, clinical characteristics, genetic variants, and treatment access among patients with HAE in the three Baltic states of Estonia, Latvia, and Lithuania. Their findings suggest that population-specific databases linking phenotypes and genotypes may help reduce diagnostic delays and expedite access to standard treatments, such as C1 inhibitor replacement therapy.

## At the forefront of clinical practice

Advances in understanding disease mechanisms and diagnostic techniques have led to the identification of rare genetic variants that were previously overlooked in clinical practice. Lipopolysaccharide-responsive beige-like anchor protein (LRBA) deficiency is primarily characterized by chronic immune thrombocytopenia, autoimmune hemolytic anemia, and enteropathy. However, its clinical presentation is highly heterogenous. Hbibi et al. reported cases of LRBA deficiency in five families in Morocco and emphasized the importance of early genetic diagnosis for guiding targeted therapies, including abatacept and HSCT.

Magnesium transporter 1 (MAGT1) deficiency, also known as XMEN disease, is a rare PID/IEI associated with defective N-linked glycosylation. Gunderman et al. identified a novel *MAGT1* variant in a patient with severe atopic dermatitis, hypogammaglobulinemia, and viral skin infections. These findings suggest that MAGT1 deficiency should be considered as a potential PID/IEI in the differential diagnosis of patients with severe atopic dermatitis resistant to standard treatment.

GATA-binding protein 2 (GATA2) deficiency is caused by heterozygous loss-of-function mutations in the *GATA2* gene and is characterized by immunodeficiency, bone marrow failure, and a predisposition to myeloid neoplasms. Its clinical presentation is highly diverse, making early diagnosis difficult. reported two Japanese adolescents with novel *GATA2* variants. One patient presented symptoms resembling inflammatory bowel disease, including features like Crohn’s disease, and later developed acute myeloid leukemia. The other presented with refractory skin warts and pancytopenia and later developed myelodysplastic syndrome. These cases highlight the importance of diagnosing GATA2 deficiency before the onset of hematological abnormalities and performing HSCT as a curative treatment when appropriate.

X-linked lymphoproliferative syndrome type 1 is caused by variants in the *SH2D1A* gene and is frequently complicated by Epstein-Barr virus (EBV)-associated hemophagocytic lymphohistiocytosis (HLH), which is often fatal. Boyarchuk et al. identified a novel *SH2D1A* variant in a 1-year-old boy who presented with fatal EBV-associated HLH. Two of the boy’s siblings had also died from HLH around the same age. This report underscores the importance of early genetic testing in HLH.

Familial HLH type 3 (FHL3), caused by *UNC13D* variants, is the most common form of FHL in Asia. Xiong et al. reported the clinical and genetic characteristics of five patients with FHL3 and hypogammaglobulinemia at a single institution in China. Their findings suggest that immunoglobulin levels should also be assessed in patients with HLH.

X-linked hyper-IgM syndrome is a combined immunodeficiency caused by immunoglobulin class-switch defects and characterized by hypogammaglobulinemia and opportunistic infections. Nishikawa et al. identified a p.Arg203Ile variant in *CD40 ligand* (*CD40L)* in an 18-year-old male undergoing follow-up for hypogammaglobulinemia. In this patient, CD40L expression on activated T cells was normal; however, binding to CD40 was absent. The p.Arg203Ile variant is a hypofunctional variant that destabilizes the CD40L-CD40 interaction without affecting CD40L expression, which may explain atypical clinical presentation.

## Conclusion

This research topic highlights three key messages to our readers. First, autoimmunity lies at the core of PID/IEI. Immunodeficiency and autoimmunity are not mutually exclusive; rather, they share common mechanisms involving the breakdown of immune tolerance. Therefore, PID/IEI should be considered as a potential underlying cause of refractory allergies and autoimmune diseases.

Second, combining nationwide screening programs, such as NBS, with innovative retrospective diagnostic approaches, including genetic analysis of preserved umbilical cords, may strengthen diagnostic across generations of patients with PID/IEI.

Third, even within the same PID/IEI classification, clinical presentations may vary because of differences in the underlying genetic variants. Therefore, precise molecular diagnosis is essential for selecting the optimal targeted therapy.

